# Polyaniline Based Pt-Electrocatalyst for a Proton Exchanged Membrane Fuel Cell

**DOI:** 10.3390/polym12030617

**Published:** 2020-03-08

**Authors:** Wen-Yao Huang, Mei-Ying Chang, Yen-Zen Wang, Yu-Chang Huang, Ko-Shan Ho, Tar-Hwa Hsieh, Yu-Chun Kuo

**Affiliations:** 1 Department of Photonics, National Sun Yat-sen University, 70 Lienhai Rd., Kaohsiung 80424, Taiwan; wyhuang@mail.nsysu.edu.tw (W.-Y.H.); mychang01@mail.nsysu.edu.tw (M.-Y.C.); 2 Department of Chemical and Materials Engineering, National Yun-Lin University of Science and Technology, 123 University Road, Section 3, Douliou, Yunlin 64002, Taiwan; 3 Department of Chemical and Materials Engineering, National Kaohsiung University of Science & Technology, 415 Chien-Kuo Road, Kaohsiung 80782, Taiwan; ych@nkust.edu.tw (Y.-C.H.); thh@nkust.edu.tw (T.-H.H.); loomin17@hotmail.com (Y.-C.K.)

**Keywords:** polyaniline, vulcan carbon black, Pt-catalyst, PEMFC

## Abstract

Calcination reduction reaction is used to prepare Pt/EB (emeraldine base)-XC72 (Vulcan carbon black) composites as the cathode material of a proton exchange membrane fuel cell (PEMFC). The EB-XC72 core-shell composite obtained from directly polymerizing aniline on XC72 particles is able to chelate and capture the Pt-ions before calcination. X-ray diffraction spectra demonstrate Pt particles are successfully obtained on the EB-XC72 when the calcined temperature is higher than 600 °C. Micrographs of TEM and SEM illustrate the affluent, Pt nanoparticles are uniformly distributed on EB-XC72 at 800 °C (Pt/EB-XC72/800). More Pt is deposited on Pt/EB-XC72 composite as temperatures are higher than 600 °C. The Pt/EB-XC72/800 catalyst demonstrates typical type of a cyclic voltammograms (C-V) curve of a Pt-catalyst with clear Pt–H oxidation and Pt–O reduction peaks. The highest number of transferred electrons during ORR approaches 3.88 for Pt/EB-XC72/800. The maximum power density of the single cell based on Pt/EB-XC72/800 reaches 550 mW cm^−2^.

## 1. Introduction

With purpose to curb the evolved greenhouse gas [1,2,3] produced from burning fossil fuels, the fuel cell has been globally researched in all fields [4].

Lately studies pay attention on hydrogen-based proton exchange membrane fuel cells (PEMFCs) under the consideration of their water by-products and significant power generation with quiet and low-temperature operations. However, the main barrier to mass-producing PEMFCs is to prepare the fuel cell in a cheaper way through increasing the efficiency and durability of the loaded Pt-catalyst [5]. Other properties concerning about the gas fuel concentration flowing into the membrane electrode assembles (MEAs) also play important roles on the functioning of the PEMFCs [6], which are related to catalyst, fuel, and binder boundaries (triple-phase boundary).

Conducting carbon black (CB) is commonly applied as the both the e-transferred and catalyst-supporting materials, considering their conductivity, highly porous structure, and cheapness. Past researches paying attentions on creating small pores on CB-supports to capture and disperse the reduced Pt [7]. Most of the studies focus on loading Pt-nanoparticles on CB (Vulcan XC72). However, cathode-catalyst substrates made of CB cannot withstand the corrosion effect from the produced hydrogen peroxide and water after oxygen reduction reaction (ORR), resulting in the formation of detrimental carbon monoxide and carbon dioxide during high temperature operation [8,9,10], causing quick poisoning of the Pt nanoparticles and effectively shortening the lives of PEMFC. Prof. Joong Kee Lee proposed a gradient activation method to enhance the discharge performance and perfecting activation mechanism of the direct methanol fuel cell (DMFC) [11]. Prof. Jong Hyun Jang, etc., first tried to improve the efficiency of PEMFC by direct spraying catalyst on the Nafion membrane [12].

N-containing coverage with calcined polyaniline (PANI) on the carbonaceous surfaces demonstrated significant ORR in both acidic and alkaline media [13,14,15,16]. Additionally, the amino groups of the N-containing PANI on XC72 support can coordinate with Pt ions, resulting in the well-dispersed Pt-catalyst on the XC72 surfaces and control the pores sizes of the obtained PANI/XC72 composites [17,18].

A polyaniline(EB)/XC72 core-shell composite can be obtained by covering XC72 with polyaniline through chemical or electrochemical polymerization, which was then neutralized by ammonium water [19,20,21].

The cladding EB can prevent the carbon matrix from CO-poisoning and behave as both chelating and reducing agents for Pt ions during calcination. Prof. Xindong Wang used PANI-coated plate to improve the corrosion resistance of electrodes [22]. Simultaneously, EB can undergo carbonization during calcination, leading to formation of nitrogen-containing conducting coverage on XC72. Eventually, a Pt-catalyst with Pt elements implanted on EB-XC72 can be obtained, which can be applied to prepare MEAs with a cathode made of a Pt/EB-XC72 catalyst. Our published studies focused on obtaining a Pt-catalyst through heating in microwave radiation using either N-phenyl-p-phenylenediamine or its polymer as the N-sources and reducing agent [23,24]. Both N-containing catalyst demonstrated better ORR and high power density for the prepared single cell. However, the temperature of the microwave oven cannot be easily controlled and we were not able to constantly increase the temperature at a constant heating rate. To avoid the sudden boiling of the E (ethylene glycol)-solvent (heating medium, which can absorb microwaves and transfer to Pt ions), the Pt-catalysts were prepared in several steps of heating by microwave irradiation with 10 s for each step, resulting in the wide range of particle size distribution. Besides, the removal of the EG solvent from the obtained Pt-catalyst turned to be very difficult due to its high boiling point.

In this study, we simply use chemical polymerization (ammonium persulfate as initiator) to cover XC72 particles with PANI (EB type), which can firmly capture Pt ions through coordination and directly turned them into Pt-catalyst after high temperature calcination. Properties of Pt-implanting % on the Pt-catalyst, ORR reactivity, electrochemical behaviors of the fabricated MEA, and the power generating capacity of the resultant cell will be studied for the PEMFC prepared with Pt/EB-XC72 cathode. 

## 2. Material and Methods

### 2.1. Materials 

The ammonium persulfate (Merck KGaA, Darmstadt, Germany), hydrogen hexachloroplatinate (IV) hexahydrate (H_2_PtCl_6_·6H_2_O, Merck KGaA, Darmstadt, Germany), aniline (TOKYO KASEI KOGYO CO., Tokyo, Japan), ammonium water (J.T. Baker, Fisher Scientific, Loughborough, UK), and Vulcan XC72 (Cabot, Boston, MA, USA) were used with no further purification. 

### 2.2. Preparation of EB-XC72 

Of anilines 5 g was mixed with 50 mL water and pH of the mixture was adjusted to 1 by the addition of 1 mol/L HCl (aq), followed by the introduction of 1 g of XC72. After homogeneously stirring for 10 min, 10 g of ammonium persulfate (APS) was added to initiate the polymerization and the reaction was continuously carried out for 30 min. PANI/XC72 was collected by filtration and the filter cake was rinsed alternatively with de-ion water and acetone to remove the unreacted monomers and oligomers. The filter cake was then mixed with excess 1 mol/L ammonium water and stirring for 24 h before EB-XC72 was collected by centrifugation. The EB-XC72 was dried in vacuum oven at 60 °C for 6 h.

### 2.3. Preparation of Pt/XC72 and Pt/EB-XC72 

Of the EB-XC72 composite or neat XC72 80 mg was first put into a vial followed by the addition of 0.56 mL of 0.048 mol/L H_2_PtCl_6_ (aq) and the mixture was under sonication until uniform and was neutralized with 9 mL, 1 mol/L NaOH(aq) until pH value reached 11 and subjected to calcination at different temperatures (500, 600, 800 and 900 °C) in a high temperature oven. The heating rate was kept at 10 °C/min in Ar atmosphere and the highest temperatures was maintained for 1 h isothermally.

### 2.4. Characterization

#### 2.4.1. WXRD (Wide Angle X-Ray Diffraction) 

A copper target (Cu-Kα) Rigaku x-ray source with a wavelength of 1.5402 Å was used for x-ray diffraction. The scanning angle (2θ) started from 5° to 40° with a voltage of 40 kV and a current of 30 mA, operated at 1° min^−1^.

#### 2.4.2. TEM (Transmission Electron Microscopy) 

Samples taken photos by field emission transmission electron microscope, HR-AEM (HITACHI FE-2000, Tokyo, Japan). They were first dispersed in acetone and put on carbonic-coated copper grids dropwise before subjecting to the emission.

#### 2.4.3. SEM (Scanning Electron Microscopy)

The sizes and morphologies of all samples were characterized by SEM (field emission gun scanning electron microscope, AURIGAFE, Zeiss, Oberkochen, Germany

#### 2.4.4. TGA (Thermogravimetric Analysis) 

The residue weights of thermal degradation of all samples were recorded by TGA (TA SDT-2960, New Castle, DE, USA) thermograms. The amount of Pt loaded were characterized by the residual weights at 800 °C at 10 °C min^−1^ with purging air.

#### 2.4.5. Raman Spectroscopy

The Raman spectra of all samples were obtained from a Raman spectrometer (TRIAX 320, HOBRIA, Kyoto, Japan).

#### 2.4.6. EDS (Energy Dispersive X-Ray Spectra)

The EDS of various Pt-catalysts were obtained from an XL-40EFG, Philips (Eindhoven, the Netherland). The samples were coated with gold before measurement.

### 2.5. Electrochemical Characterization 

The cyclic voltammetry method was used to determine the active electrochemical surface area of the catalyst supports in the electrode. The performance of the electrocatalyst support was tested with a three-electrode system. The square working electrode with an area of 1.5 cm^2^ was prepared as follows. Ag/AgCl and a platinum wire were used as the reference and counter electrode, respectively. An electrochemical test was carried out in a potentiostat/galvanostat (Autolab-PGSTAT 30 Eco Chemie, Utrecht, Netherland) in 0.5 mol/L H_2_SO_4_ solution and cyclic voltammograms (C-V) were obtained with a scanning potential from 0.0 to 1.4 V at a sweeping rate of 10 mV s^−1^. The catalyst ink was prepared by mixing 3 mg support powder in isopropanol and stirred until it became uniform. Subsequently, 5% Nafion solution was added into the mixture as binder and the mixture was ultrasonicated for 1 h, the obtained ink was uniformly cast on the carbon paper for the C-V test. 

The polarization curves of all Pt- catalysts were measured using a rotating-disk electrode (RDE) operated at 900, 1200, 1600, 2500 and 3600 rpm for in O_2_-saturated 0.5 mol/L H_2_SO_4_, respectively. The ORR currents were recorded within the measured voltage range (0.0–1.0 V) at 1600 rpm.

### 2.6. MEA Preparation

A Nafion^®^ 212 sheet purchased from Ion Power Inc. (New Castle, DE, USA), was used as the proton exchange membranes. In order to remove the surface organic impurities and to convert the membranes into protonated (H^+^) form, the Nafion-212 (4 × 4 cm^2^) membrane was treated at 70 °C in 5 wt % H_2_O_2_ aqueous solution for 1 h, followed by submerging in 1 mol/L H_2_SO_4_ solution for 1 h and subsequently the treated membranes were dipped in distilled water for 15 min and stored in de-ionized water. The catalyst inks were prepared by mixing 20 mg of prepared Pt-catalyst powders in isopropanol and mechanically stirred until it became uniform before 5% Nafion solution was added. Eventually, the catalyst mixture was ultra-sonicated for 1 h followed by coating on both side of the treated Nafion sheet dropwise as anode and cathode electrodes (2 × 2 cm^2^), respectively and hot-pressed at 140 °C with a pressure force of 70 kg cm^−2^ for 5 min to obtain the MEA.

### 2.7. Single-Cell Performance Testing

The MEA was installed in a fuel cell test station for testing using the single-cell test equipment (model FCED-P50; Asia Pacific Fuel Cell Technologies, Ltd., Taichung, Taiwan). The active cell area was 2 × 2 cm^2^. The temperatures of anode, cell, and cathode and humidifying gas were all maintained at around 70 °C The flow rates of anode input H_2_ and the cathode input O_2_ fuels were set at 100 and 200 mL min^−1^, respectively. To test the electrochemical performance of Pt-catalysts, both polarization curves (I-V) and output powers were recorded.

## 3. Results and Discussion

### 3.1. XRD Pattern of the Electrocatalyst Electrode Materials

**Figure 1:**
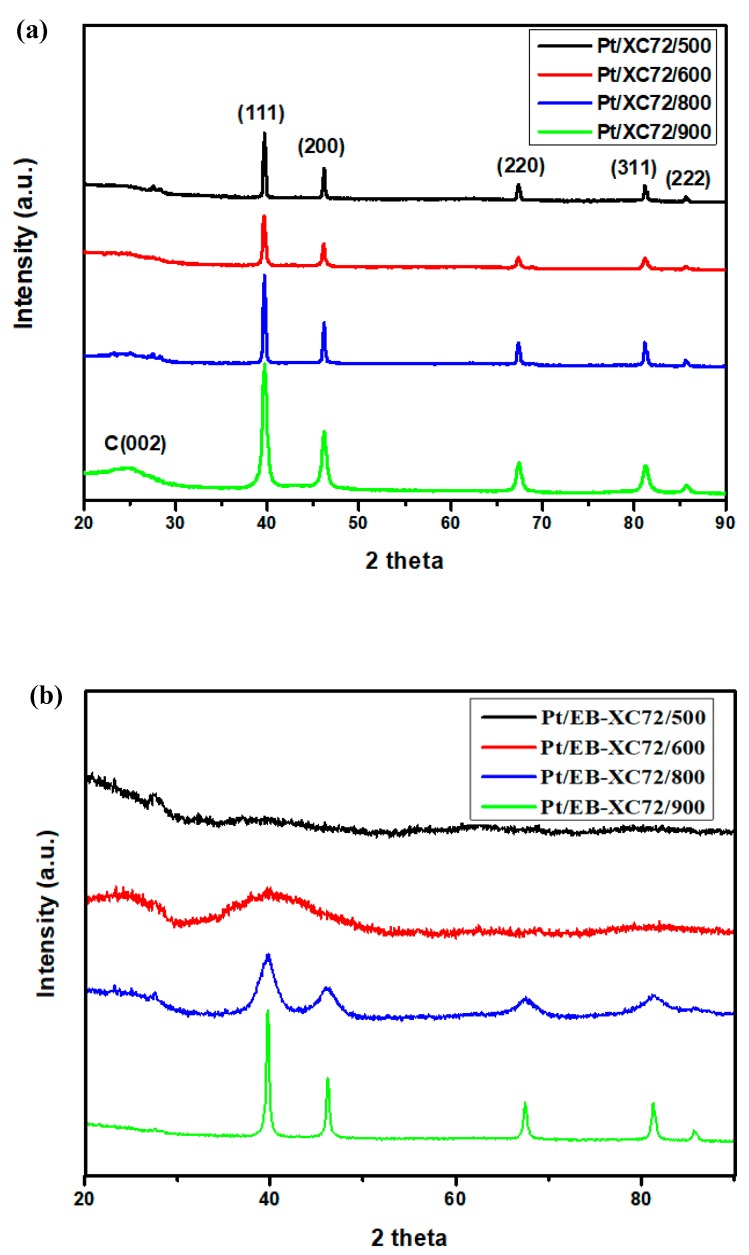
X-ray diffraction patterns of Pt-catalysts treated at various temperatures. (**a**) Pt/XC72 (**b**) Pt/EB-XC72.

The XRD patterns of Pt-crystalline loaded on all supports are demonstrated in Figure 1. The Pt deposited on the neat XC72 surface demonstrated the characteristic diffraction peaks (FCC) of Pt crystalline at (111), (200), (220), (311) and (222), respectively, revealing the Pt can be obtained easily via high temperature calcination in the absence of any reducing agent and the prepared Pt-crystals own similar type of diffraction pattern like ordinary Pt elements. The sharp diffraction peaks found in Figure 1a are related to the perfect crystalline structure of the obtained Pt-catalyst. The carbonized crystalline peak around 26° ((002) plane of carbon crystals) grows with calcination temperature due to the gradual ordering of the XC72 crystalline structure. After covering the XC72 with EB-polyaniline (PANI), the diffraction peaks of the obtained Pt-catalysts became much broader when the calcined temperatures were below 800 °C according to Figure 1b, indicating the crystallinity of the Pt-NPs loaded on the EB-XC72 was very low. Surprisingly, no significant crystalline form of Pt can be found for EB-XC72 substrate until temperature reached 800 °C (Figure 1b). However, the perfectness of crystalline does not mean the obtained Pt-catalysts are well-dispersed on the supports, which is required for good electrode-catalysts. The EB type of PANI covering on the XC72 surfaces also went carbonization during calcination [25,26,27,28], which contributed to the formation of the broad diffraction peak of (002) plane as well. 

The mean crystallite sizes of Pt implanted on various substrates and treated at different temperatures are obtained from XRD patterns and listed in Table 1. Higher calcination temperature results in larger particle size for all substrates. Larger (>3 nm) particles were easily found for neat XC72 at all calcination temperatures and no significant large Pt-crystals can be found for EB-XC72 substrate until the temperature reached 900 °C. It seems the EB shell on the XC72 core does not provide a favorable condition for growing bigger Pt particle at lower calcination temperatures. However, larger Pt-crystals do not guarantee higher loaded Pt %. Total amount of implanted Pt can be decided by the residue weight of the corresponding TGA thermogram.

(1) $$d=\frac{k\lambda }{\beta \cos\theta }$$

where k is a coefficient (0.9), λ is the wavelength of the X-rays (0.1541 nm for CuKα), β is the full-width half-maximum (FWHM) of the respective diffraction peak measured at 2θ (in radians), and θ is the diffraction angle of the peak in degree.

**Scheme 1:**
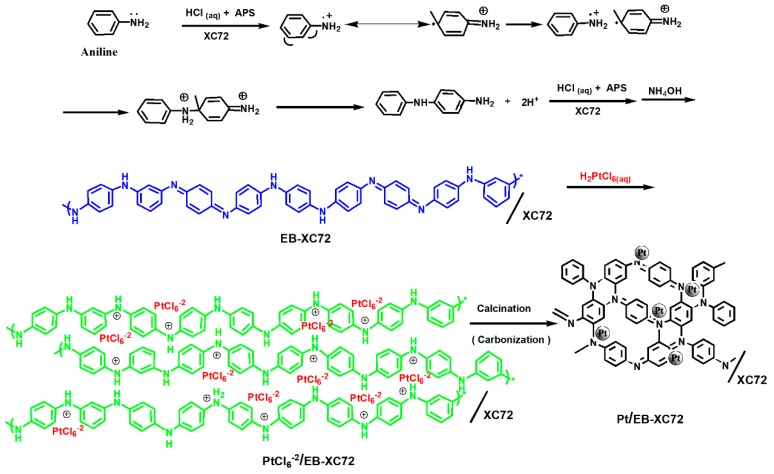
Formation mechanism of Pt/EB-XC72.

**Scheme 2:**
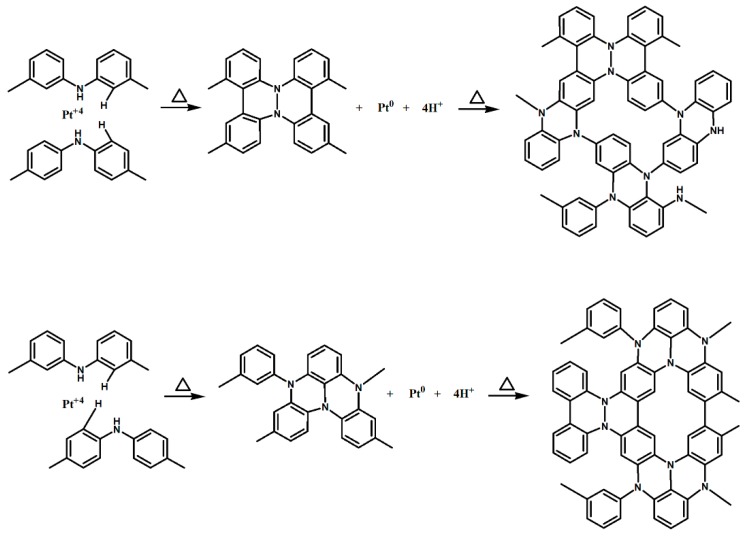
Calcination mechanism of Pt/EB-XC72

PANI (EB) can not only behave as an N-source but its amine groups are good reducing agents for Pt ions during calcination as depicted in Scheme 1. During the high temperature redox reaction between EB and Pt ions, cyclization between EB molecules [25,26,27,28] could occur in accordance with Scheme 2, leading to the formation of ladder-like structure, which can improve the planarization of N-doped carbon networks through crosslinking (carbonization). Eventually, most of the N-atoms of EB were embedded in the carbon planes, which can increase the oxygen capturing capability for higher power generation.

### 3.2. Transmission and Scanning Electron Microscopy (TEM and SEM)

**Figure 2:**
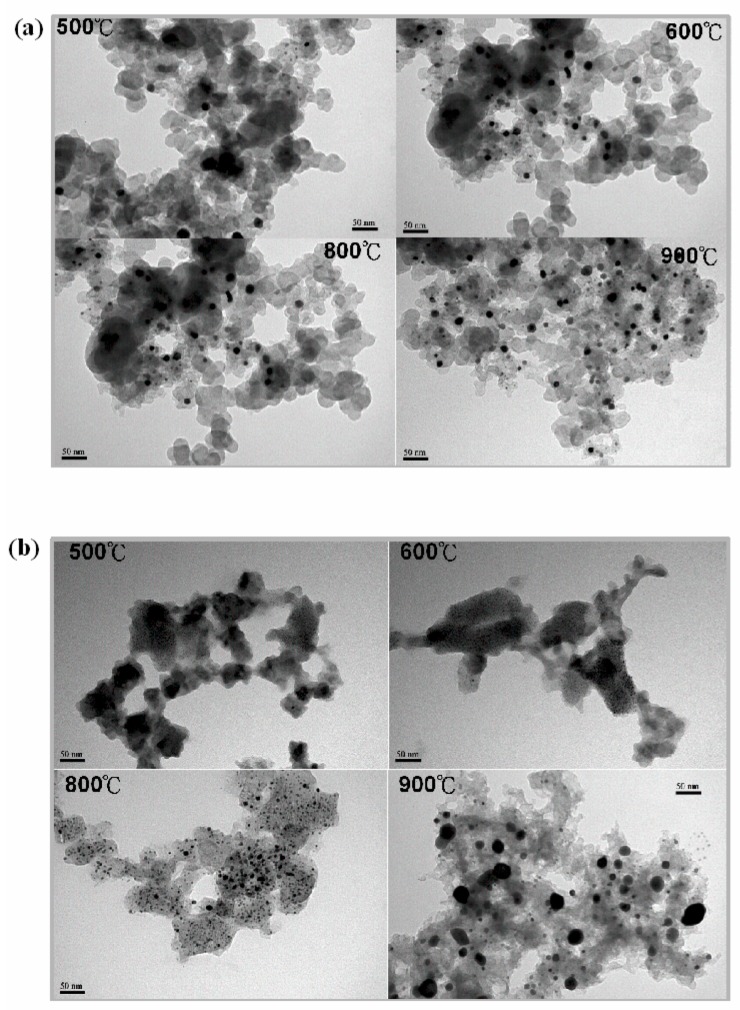
TEM images of various Pt-catalysts treated at different temperatures: (**a**) Pt/XC72 and (**b**) Pt/EB-XC72.

The particle and size distribution of Pt-NPs derived from calcination on various supports could be roughly estimated from the TEM micrographs demonstrated in Figure 2, which revealed the locations and degree of segregation of the implanted Pt-NPs. Obviously, significant, large Pt-NPs could be easily seen for Pt/XC72 when temperature was higher than 500 °C (Figure 2a). Whereas, after XC72 was covered by EB, no significant Pt-NPs could be found at temperature below 800 °C (Figure 2b). The averaged Pt-crystal size at each calcination temperature could also be obtained from the X-ray diffraction peak ((220) plane of Figure 1) based on the FWHM method, which are listed in Table 1. The mean Pt-crystal size grew from 3.01 (500 °C) to 5.20 nm (900 °C) on the surface of neat XC72 support, which is roughly similar to the TEM micrographs in Figure 2a. Table 1 illustrates that the sizes of Pt-NPs implanted on EB-XC72 substrate are below 1 nm until the temperature was close to 800 °C and no Pt-crystals could be found below 600 °C. The results derived from Table 1 reported almost the same trend that was found in the TEM micrographs of Figure 2b.

After EB molecules covered the surface of XC72, they could be protonated by H_2_PtCl_6_ acid and most of the Pt ions (in the form of PtCl_6_^2−^) could be attracted by the protonated N^+^ by coulombic interaction (Scheme 1). Consequently, Pt ions could stay on the EB-XC72 surface (Figure S1) before and after the calcination. Eventually, covering XC72 with EB did result in more Pt-loading, which will be confirmed by the TGA thermograms. 

**Figure 3:**
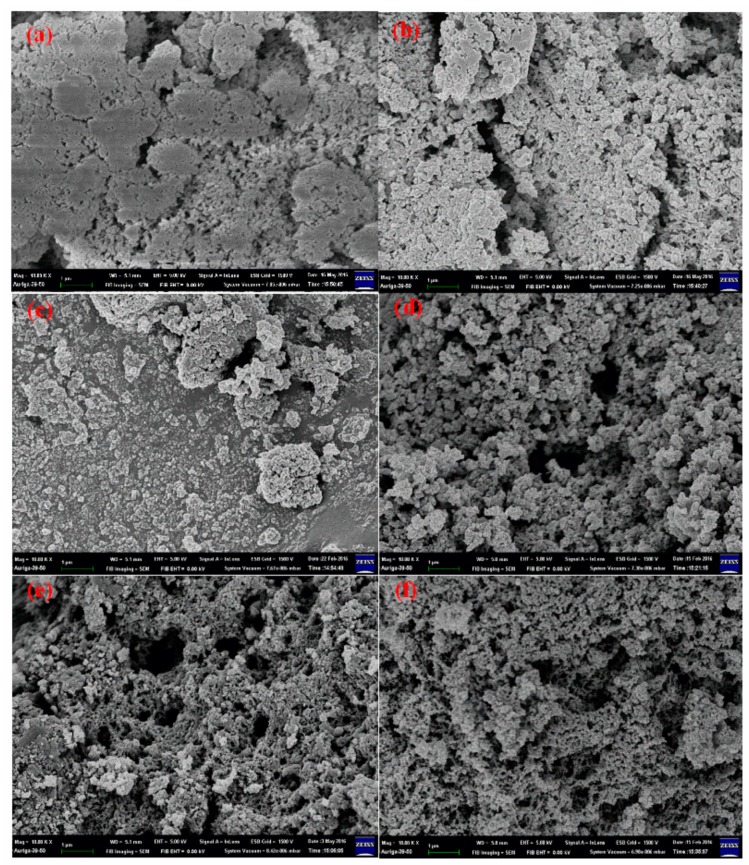
SEM images of Pt/EB-XC72 treated at different temperatures. (**a**) Neat EB-XC72, (**b**) PtCl_6_^2−^/EB-XC72 at RT, (**c**) 500 °C, (**d**) 600 °C, (**e**) 800 °C, and (**f**) 900 °C.

The size and distribution of the surface pores of the catalysts are also decisive points to the yield of the power produced by oxidation of H_2_ and the reduction of O_2_. Both of the reactants are in gas states, which can only be catalyzed by diffusing into the pores of the solid-catalyst. Therefore, we need to create as many pore as possible to accommodate the gas fuels. Neat XC72 provide significant pores on the surface whose size and distribution did not change after Pt-implantation at high temperature calcination according to their SEM micrographs demonstrated in Figure S2. Additionally, part of the surface pores was covered by the EB molecules after polymerization and followed neutralization by ammonium water (Figure 3a). These covered pores by EB can still not recover after complexing with H_2_PtCl_6_ (Figure 3b) until the calcination temperature reached higher than 600 °C (Figure 3c–f). We also understand from Figure 1 that most of the implanted Pt-NPs had very low crystallinity and small sizes when calcination temperatures were below 800 °C for Pt/EB-XC72. Based on the information provided by the SEM micrographs, we will see the electrochemical performance of the fabricated MEA and single cell can only be improved by calcination at temperatures higher than or equal to 800 °C. 

### 3.3. Thermo Gravimetric Analysis (TGA)

**Figure 4:**
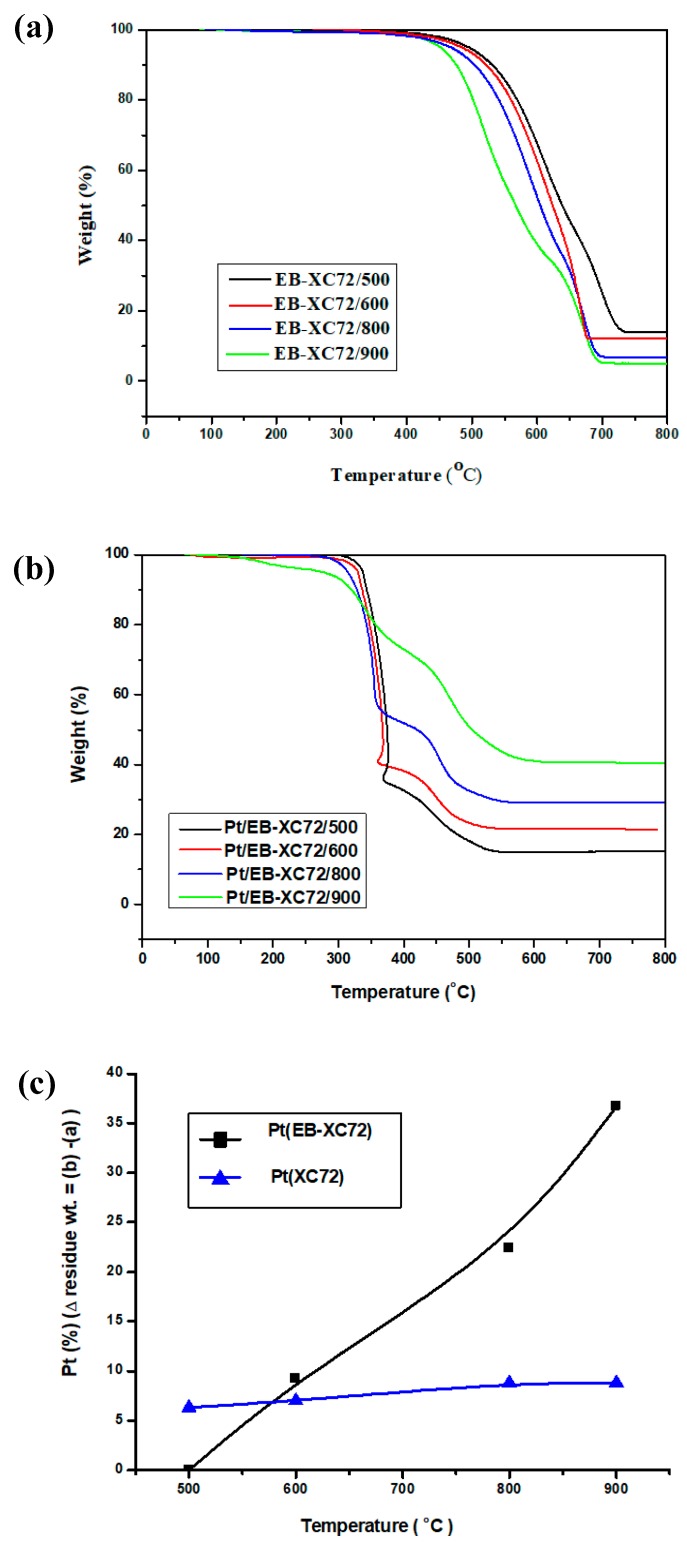
TGA thermograms and Pt-loading % of Pt/EB-XC72 treated at various temperatures. (**a**) EB-XC72, (**b**) Pt/EB-XC72, and (**c**) Pt-loading % of various Pt-catalysts.

The Pt-catalyst was based on the XC72 carbon black covering with EB ahead of calcination. Therefore, the degradation behavior and residue weight of the neat EB-XC72 matrix needs to be understood if we want to realize the exact quantity of the implanted Pt-NPs. Figure S2a clearly demonstrates that neat XC72 went on to be fully devastated after 700 °C. However, XC72s that have calcined at temperatures below 700 °C (500 °C and 600 °C) demonstrated a different degree of residue weights in the second calcination in TGA analysis, in other words, the high residue weight of the Pt/XC72 catalysts that obtained at either 500 °C or 600 °C (Figure S2a) did not mean high Pt yield. Consequently, a significant residue weight drop is found for both XC72/800 and XC72/900 due to the entire degradation of the XC72 matrix experiencing the highest degradation temperature (700 °C) before the TGA analysis. Similar significant residue-weight drops were also found for both EB-XC72/800 and EB-XC72/900, as shown in Figure 4a. Besides, not all the organic parts of the Pt-catalysts degrade completely during calcination. EB molecules themselves went on intermolecular crosslinking and carbonization during calcination [25,26,27,28] and contributed to part of the residue weight. The degradation of XC72 might not be complete after 700 °C when it was protected by EB (EB-XC72) during calcination. 

Therefore, the real Pt-loading % on XC72, EB-XC72 substrates can only be obtained by the difference of residue weight with and without Pt-implantation treated at the same temperature. In other words, the curve of Pt (on EB-XC72 substrate) in Figure 4c was obtained by subtracting the residue weight of Figure 4a with that of Figure 4b. Additionally, curves of Pt (on XC72 substrate) in Figure 4c were obtained from Figure S3a,b. Figure 4c revealed almost no Pt was present on the EB-XC72 surface when temperature was at 500 °C. The Pt-loading % rose with calcination temperature and up to 36.6% (Figure 4c) for Pt/EB-XC72 when it was calcined at 900 °C. The Pt-loading % on XC72 did not change a lot with calcination temperatures according to Figure 4c. There were less than 10% of Pt loaded on XC72 surface and only slightly increased with temperature. These results were very similar to the conclusions we obtained from TEM micrographs in Figure 2.

### 3.4. Raman Spectroscopy

**Figure 5:**
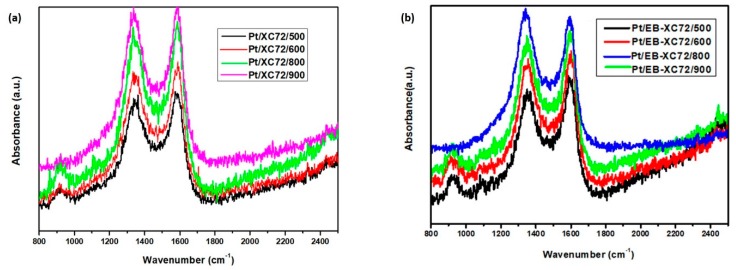
Raman spectra of (**a**) Pt/XC72 and (**b**) Pt/EB-XC72.

D–band (1350 cm^−1^) of Raman spectrum represents the sp^3^-bonding of the carbon and G-band for sp^2^-bonding (1590 cm^−1^) and the intensive ratio of them (I_D_/I_G_) can be used to estimate the degree of ordering of materials after calcination. The Raman spectra illustrated in Figure 5 were obtained from Pt/XC72 and Pt/EB-XC72 treated at various temperatures. Their I_D_/I_G_ values are listed in Table 2, which indicates the degree of ordering of both Pt-XC72 and Pt/EB-XC72 decreased with calcination temperature and Pt/EB-XC72 decreased at a faster speed. Pt/EB-XC72 always demonstrated a lower degree of ordering than PT/XC72 when treated with at the same temperature. It seems that more Pt-loading obtained by calcination would also cause the loss of crystallinity (order) of the catalyst supports. In other words, Pt-reduction by high temperature calcination was performed at the expense of losing the degree of ordering of the supporting matrix. However, the decreasing ordering does not mean the higher weight loss by calcination when comparing Figure 4b with Figure S2b, we understand that XC72 cores can be protected by EB shells during calcination.

### 3.5. Energy Dispersive X-Ray Spectroscopy (EDS)

**Figure 6:**
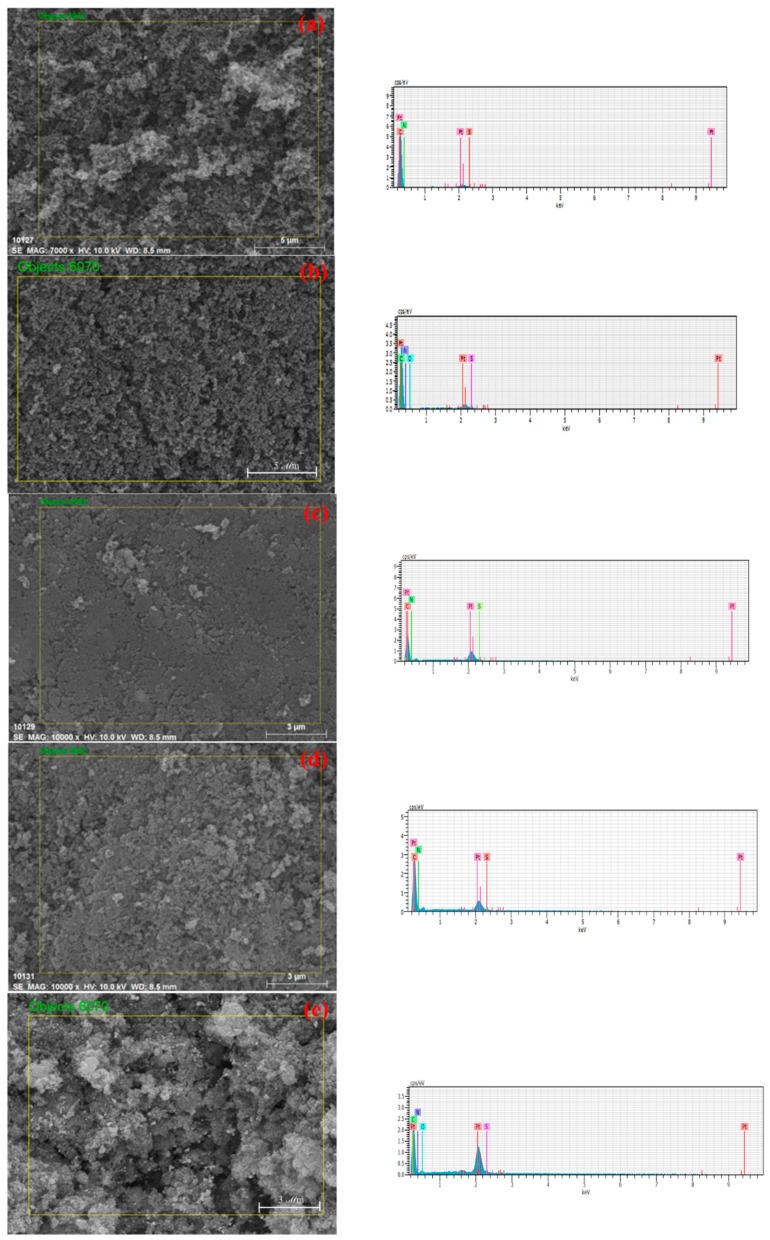
SEM micrographs and EDS of (**a**) Pt/XC72/500, (**b**) Pt/XC72/800, (**c**) Pt/EB-XC72/500, (**d**) Pt/EB-XC72/800, and (**e**) Pt/EB-XC72/900.

The atomic ratios (C, N, and Pt) obtained from EDS are illustrated Figure 6 and listed in Table 3 for Pt-catalysts treated at various temperature. Although the exposed area to x-ray did not cover the entire sample, the ratios of each type of element present after calcination can still be roughly estimated by EDS. The presence of N– at all calcination temperatures (Table 3) proved the presence of N-doped Pt-catalyst after calcination for Pt/EB-XC72. No significant N– could be found for Pt/XC72 after calcination and the presence of O– in the XC72 matrix at 800 °C was related to oxidation, which did not result in significant destruction of the matrix structure, comparing the SEM micrographs illustrated in Figure 6a,b. However, Pt/EB-XC72 also underwent oxidation when the calcination temperature reached 900 °C (Table 3) but the oxidation caused a significant destruction of the matrix according to the SEM micrographs in Figure 6c–e, which could possibly change the electrochemical properties of the obtained Pt-catalyst and will be discussed in the section of the electrochemical analysis. 

### 3.6. Electrochemical Analysis 

#### 3.6.1. Cyclic Voltammetry (C-V)

The electrochemical activities of some Pt-catalysts with significant electrochemical activities were checked by measuring the corresponding C-V by sweeping the potential, scanning at 50 mV s^−1^.

**Figure 7:**
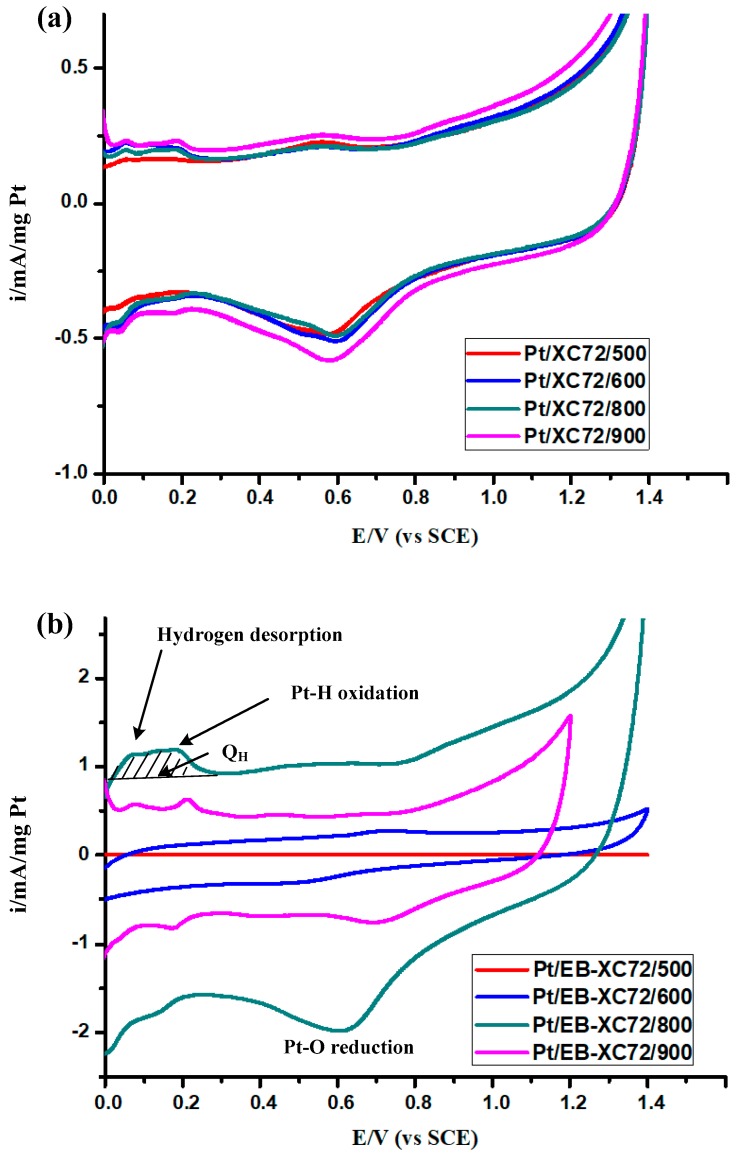
The cyclic voltammetry (C-V) curves of Pt-catalysts treated at different temperatures: (**a**) without EB and (**b**) with EB.

The catalyzing capability of the implanted Pt particles can be enhanced when they are well-dispersed to expose more surface area to the gas fuel. Definitely, some of the active surface of the Pt-catalyst will be sacrificed when implanting on the conducting support to transport the electrons. Consequently, the Pt needs to be prepared in a way that particles can be well-separated from each other to uncover as many active sites as possible to perform the redox reaction. The electrochemical active surface area (ESA) and specific ESA (ECSA) listed in Table 4 were calculated from the corresponding C-V curve in Figure 7 and residue weight obtained from the corresponding TGA thermogram of each type of Pt-catalyst. When Pt was loaded on the neat XC72 by calcination, their C-V curves illustrated an insignificant relationship with calcination temperatures. Almost the same C-V curves were found for different treating temperatures in Figure 7a. Figure 7b revealed higher hydrogen oxidation charge (Q_H_: the integration area of hydrogen desorption peak and Pt–O reduction area [29]) for Pt/EB-XC72/800, oxidation peaks in the C-V curves.

It seems Pt/EB-XC72/800 demonstrated higher ESA or ECSA than Pt/XC72 than the rest of the two Pt-catalysts according to Table 4, indicating it owns a more active surface area. Therefore, we understand more O_2_ was captured and a more active electrochemical active area was present on the surface of Pt/EB-XC72/800. However, when temperature rose up to be 900 °C, the redox area of the C-V curve and ECSA of Pt/EB-XC72 decreased drastically, resulting from the possible thermal degradation (Figure 4) of the conducting matrix (either XC72 or EB) at such a high temperature. 

No significant ORR peaks for Pt/EB-XC72 treated below 800 °C were found in Figure 7b although hydrogen was desorbed and oxidized, indicating ORR effectively slow down the entire redox reaction and the power generation.

#### 3.6.2. Linear Sweep Voltammetry (LSV) and Koutecky-Levich (K-L Plot)

From the above discussions about C-V curves and ECSAs, we understand that Pt/EB-XC72/800 owns the biggest possibility to enhance the ORR [32,33].

**Scheme 3:**
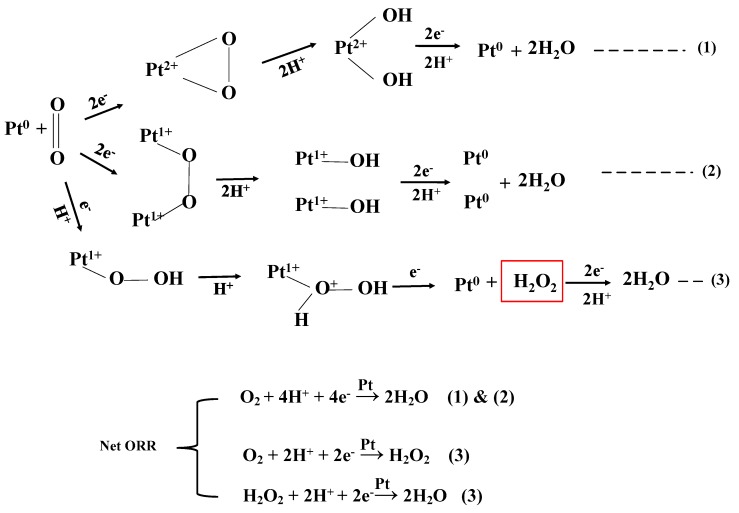
Possible oxygen reduction reaction mechanisms in the cathode.

In the anode hydrogen oxidation reaction (HOR), H_2_ gas can experience full oxidation easily by few Pt. In the cathode, the complete reduction of one O_2_ takes four protons, which are provided through the solid electrolyte in the middle of MEA in accordance with Scheme 3, which consumes either one or two Pt-catalyst (Step (1) or (2)) embedded in the cathode. The C-V curve of Pt/EB-XC72 revealed significant Pt–O reduction peak, which can represent three types of Pt–O bonding in the first reaction of Steps (1)–(3). For steps (1) and (2), the O–O (peroxide) dissociates before Pt–O and combines with H^+^ into O–H, which then converts to H_2_O in the further reduction reaction. In Step (3), the Pt–O breaks before O–O and leads to the formation of the intermediate H_2_O_2_. The presence of the additional step significantly delays the ORR in the cathode. Additionally, the retarding supply of O_2_ due to the concentration or diffusion polarization will force them to take different routes of reduction and lead to the formation of H_2_O_2_ in the additional intermediate step [34] as depicted in Step (3) of Scheme 3. Step (3) illustrates the retardation of the reduction reaction comes from the delaying supply of protons and electrons, which produces the H_2_O_2_ by-product. Even though the intermediate H_2_O_2_ will go on a further reduction into water eventually, less power will generate. Therefore, the ORR becomes the rate determining step and the improvements on ORR can effectively increase the power generation of PEMFCs.

**Figure 8:**
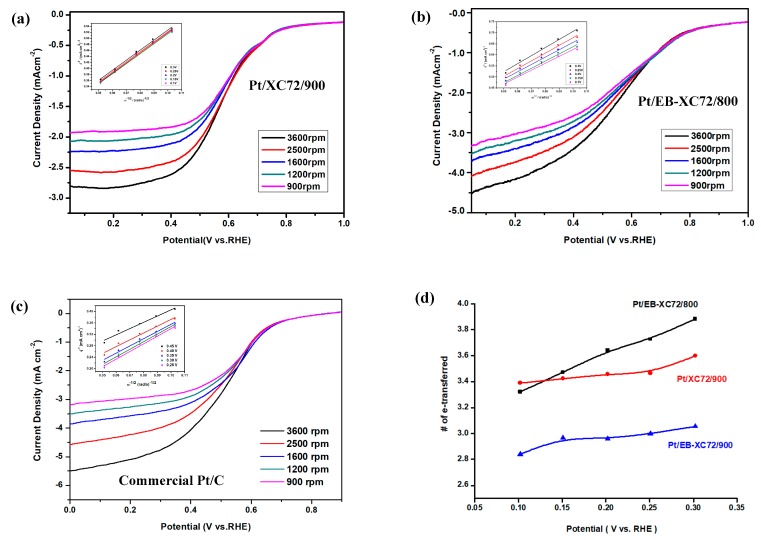
Linear sweep voltammetry (LSV) curves and Koutecky-Levich (K-L) plots of various Pt-electrodes. (**a**) Pt/XC72/900, (**b**) Pt/EB-XC72/800, (**c**) commercial Pt/C at different rotating rates, and (**d**) number of electrons transferred during ORR.

The ORR can be improved if the solid electrolyte can transport and provide protons at a faster speed, which is dependent on the conductivity of the electrolyte membrane. Larger ECSA of the Pt-catalysts, which are implanted on the conducting matrix (substrate) is another important factor that can be used to improve ORR, which can be fulfilled by loading the Pt-NPs in a well-dispersed way on the XC72 surfaces. Another important issue that is directly related to ORR is the polarization of the O_2_ supply, which is controlled by the concentration difference and the diffusion speed of the O_2_. Therefore, the straight way to illustrate the ORR of the Pt-catalyst is to measure its polarization curves when coated on the GC electrode at different disk rotation rates of the electrode (RDE) with the LSV approach. We chose only the samples demonstrating better electrochemical behavior for LSV measurements. The LSV curves of Pt/XC72/900, Pt/EB-XC72/800, and commercial Pt/C are illustrated in Figure 8a–c, respectively. The LSV curves of Pt/EB-XC72/800 are very close to that of commercial Pt/C. Reduced current densities of Pt/XC72 shift from 1.8 to 2.6 mA cm^−2^ when electrode-rotating speeds was increased from 900 to 3600 rpm (Figure 8a). For each LSV curve, the reduced current density is dependent on the input O_2_ concentration in the cathode in the initial stage (1.0–0.7 V), which is called kinetic control. Its current (kinetic current: I_kin_) almost remains the same at different rotating speeds. When the potential is declined, the reduced currents significantly depend on the rotating speeds and increased with them, which is called diffusion current (I_dif_), during which the current depends on the diffusion rates of O_2_. The diffusion-controlled stages (0.15–0.4 V) can be used to plot the K-L lines at the same voltage, which can be applied to estimate the numbers of electrons transferred when oxygen undergoes a reduction (ORR). The concentration and diffusion delay (polarization) result in the insufficient supply of O_2_ fuel in the cathode, forcing the reduction reaction to take one more step, which also delays ORR and H_2_O_2_ is the intermediate product.

The LSV curves used to construct the K-L plot (inset figures) are demonstrated in Figure 8, which were then used to calculate the numbers of the electron transfer during ORR at different potentials, listed in the 5^th^ column of Table 4.

The maximum number of transferred electrons during ORR for Pt/XC72/900 was 3.58 and that of Pt/EB-XC72/800 was 3.88 based on Figure 8d and Table 4, which was only slightly lower than the complete ORR that four electrons were transferred. In other words, less H_2_O_2_ were produced and ORR was more completed when Pt/EB-XC-72/800 was the loading catalyst in the cathode. When calcination temperature was increased to 900 °C, the e-transferred number decreased to 3.05 for Pt/EB-XC72 catalyst referring to Table 4 and Figure 8d. The lower e-transferred numbers comes from the smaller ECSA for Pt/EB-XC72/900 although more Pt-catalyst were implanted on the XC72, indicating the obtained Pt-catalyst were accumulated into bigger particles and less surface area were available to the O_2_ gas, which was confirmed by the TEM micrograph demonstrated in Figure 2b. Actually, similar aggregated Pt-NPs were also found for neat XC72 matrix after calcination, which illustrated significant bigger particles in their TEM micrographs of Figure 2a and lower e-transferred numbers (3.58) than that of Pt/EB-XC72/800 (3.88).

#### 3.6.3. Single Cell Performance Analysis

**Figure 9:**
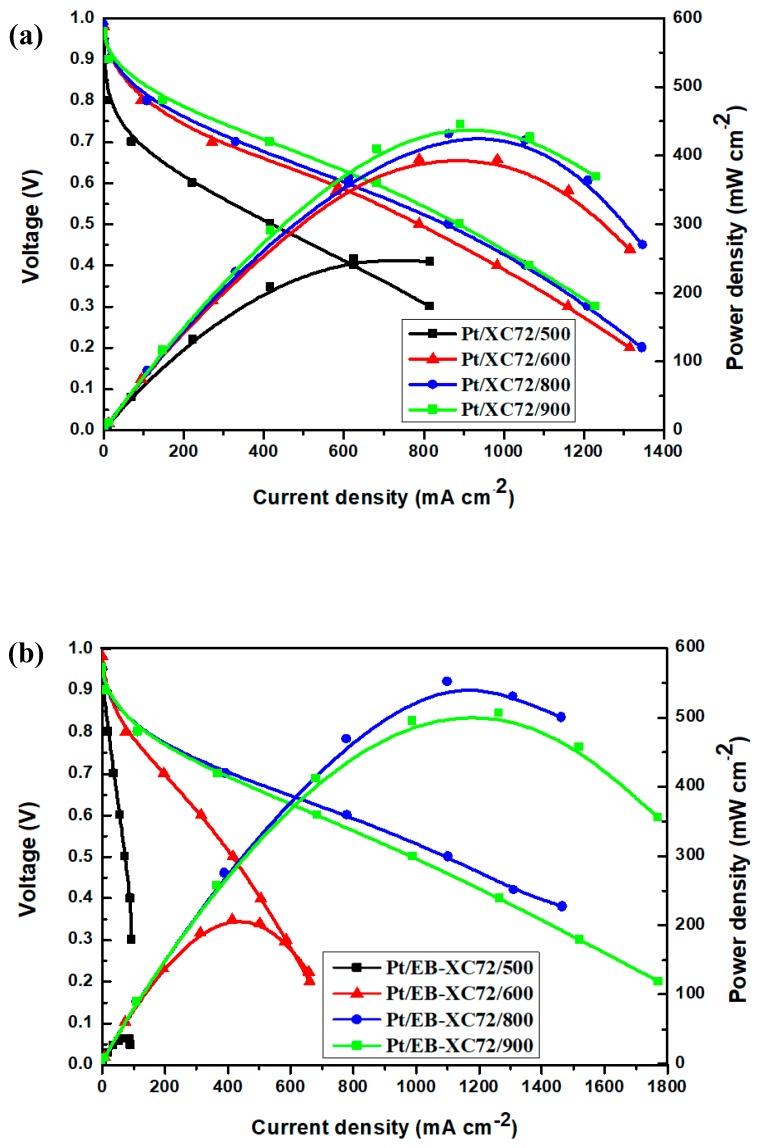
Electrocatalytic polarization and power density curve of a proton exchange membrane fuel cell (PEMFC) based on (**a**) Pt/XC72 and (**b**) Pt/EB-XC72 catalyst supports.

MEAs prepared from Pt/XC72 and Pt/EB-XC72 as cathode electrodes were assembled into single cells and their current density, voltage and power density are illustrated in Figure 9a,b, respectively. Pt-catalyst prepared by neat XC72 demonstrated maximum power density (P_max_) over 400 mW cm^−2^ when calcined at a temperature higher than 600 °C as shown in Figure 9a. However, the P_max_ increased insignificantly after 800 °C (450 mW cm^−2^), which could be understood from the slight increase of implanted Pt % in Figure 4c. For a single cell with the Pt/EB-XC72 cathode, almost no power density was generated when prepared by calcination at 500 °C due to the coverage of EB molecules on the surface of Pt/EB-XC72 cathode and no O_2_ gas can be trapped by catalyst as seen in Figure 3c. Lots of pores were created with temperatures increasing from 600 to 800 °C according to Figure 3c–f and P_max_ increased (Figure 9b) from 200 to 550 mW cm^−2^ (Pt/EB-XC72/800) higher than that of Pt/XC72/900. The better performance of a single cell with Pt/EB-XC72/800 cathode results from higher degree of Pt–H oxidation and more finely dispersed by the size of the voltammogram (Figure 7b). Besides, a higher number of e-transferred with a higher degree of ORR shown in Figure 8d also contributed the higher P_max_ of Pt/EB-XC72/800. However, the P_max_ decreased to around 500 mW cm^−2^ when temperature was elevated to 900 °C according to Figure 9b. The oxidation on Pt/EB-XC72 at 900 °C, which was already confirmed in the discussion of EDS, could bring in the destruction of the matrix and less power was generated.

## 4. Conclusions

The basic state of polyanilines (EB type) were prepared directly on the surface of XC 72 to firmly capture more Pt ions through the neutralization with hexachloroplatinic acid, followed by calcination to become the Pt-catalyst. EB was used as the nitrogen source polymerized on the carbon black, which was then calcined at the temperature of 800 °C in the argon atmosphere to obtain the N-doped Pt-catalyst. 

The electrochemical performance of the single cell made of the composite Pt-catalyst was analyzed. The PANI/XC72 core-shell composite was able to chelate and capture the Pt-ions before calcination. The shell EB also became an effective reduction agent during calcination. X-ray diffraction spectra demonstrated Pt particles were successfully obtained on the EB-XC72 composite matrix when the calcined temperature was higher than 600 °C. However, Pt-nanoparticles could be loaded at a temperature lower than 500 °C on the neat XC72 substrate. Micrographs of the TEM and SEM illustrate the affluent, smallest Pt nanoparticles were uniformly distributed on the EB-XC72 matrix calcined at 800 °C (Pt/EB-XC72/800) in the argon atmosphere. Based on the residue weights obtained from the TGA thermograms, we realized more Pt were deposited on the Pt/EB-XC72 composite compared with the neat XC72 matrix as temperatures were higher than 600 °C. The Pt-catalysts with all substrates demonstrated the typical type of C-V curve of the Pt-catalyst, illustrating clear Pt–H oxidation and Pt–O reduction peaks. The electrochemical active surface area of Pt/EB-XC72/800 was found to be much higher than that of either Pt/EB-XC72/900 or Pt/XC72/900. The highest number of transferred electrons during ORR approached 3.88 for Pt/EB-XC72/800, higher than that of either Pt/EB-XC72/900 (3.05) or Pt/XC72/900 (3.58). The maximum power density of the single cell based on Pt/EB-XC72/800 reached 550 mW cm^−2^ compared with that of Pt/XC72/900 (439 mW cm^−2^), indicating Pt-particles loaded on XC72 covered with EB could effectively improve the ORR in the cathode and become a promising material for preparing PEMFC with higher power generation. 

## Figures and Tables

**Table 1:** Mean crystal size ^a^ (nm) of Pt loaded on different substrates.

	Substrate	XC72	EB-XC72
Temperature (°C)			
**500**		3.01	-
**600**		4.42	-
**800**		4.86	1.81
**900**		5.20	3.86

a: The average implanted Pt crystal size for each electrocatalyst electrode material is calculated by the Debye–Scherrer equation based on the X-ray diffraction (220) plane of Pt crystalline (Figure 1).

**Table 2:** I_D_/I_G_ value of various Pt-catalysts.

	500 °C	600 °C	800 °C	900 °C
**Pt/EB-XC72**	0.93	0.95	1.01	1.06
**Pt/XC72**	0.90	0.90	0.91	0.95

**Table 3:** Atomic % (Energy Dispersive X-Ray Spectroscopy (EDS)) of Pt-catalysts calcined at different temperatures.

	*T* (°C)	500		800		900
Element		Pt/XC72	Pt/EB-XC72	Pt/XC72	Pt/EB-XC72	Pt/EB-XC72
**C**		99.23	82.75	97.04	86.32	86.25
**N**		0.00	15.31	0.00	10.72	5.11
**O**		0.00	0.00	1.93	0.00	4.96
**S**		0.28	0.54	0.40	0.51	0.34
**Pt**		0.49	1.40	0.63	2.45	3.34

**Table 4:** Particle size and electrochemical properties of Pt-catalysts.

	ESA ^a^ (cm^2^)	Pt-Weight ^b^ (%)	ECS ^c^ (m^2^ g^−1^_Pt_)	Pt Surface Area Per Unit Support ^d^ (cm^2^ mg^−1^)	No of E-transferred ^e^
**t/EB-XC72/800**	647	22.2	29.14	798.17	3.88
**Pt/EB-XC72/900**	74	36.6	2.02	41.83	3.05
**Pt/XC72/900**	2	8.7	2.29	88.55	3.58

a: ESA (cm^2^) = Q_H_ 0.21^−1^ (mC cm^−2^); Total hydrogen oxidation charge (Q_H_ (mC)) obtained from the integration area of H_2_ desorption (mAV) in C-V diagram (Figure 7) divided by scan speed (V s^−1^); 0.21: the theoretical, necessary charge to oxidize a monolayer of H_2_ on bright Pt; b: From Figure 4c; c: ECSA (m^2^ g^−1^) = ESA [Pt]^−1^, [Pt]: Weight of Pt in the GC electrode = weight of catalyst loaded on the GC electrode × Pt % obtained from the residue weights of the corresponding Pt-catalysts [30,31] in the 4^th^ column; d: To estimate the theoretical surface area of Pt per unit catalyst support, we can assume every Pt particle is separated from each other and is spherical with diameter (particle size) listed in 2^nd^ column. The residual % of loaded Pt listed in 4^th^ column is considered as the weight (W) and the density (ρ) of loaded Pt is about 21.45 g cm^−3^. The active surface area index can be estimated as the summation of surface area of each particle: $\left(W/(100-W)/\rho /\left(\left(4\pi /3\right)R^{3}\right)\right)*4\pi R^{2}=3W/\left(100-W\right)/21.45/R$; Where $\left(\left(4\pi /3\right)R^{3}\right)$ and $4\pi R^{2}$ are volume and surface area of single particle, respectively; e: Numbers of electrons transferred obtained from Figure 4c.

## Supplementary Materials

The following are available online at https://www.mdpi.com/2073-4360/12/3/617/s1.
