# Supplementary material for: Polyaniline Based Pt-Electrocatalyst for a Proton Exchanged Membrane Fuel Cell
Source: Polymers (Basel). 2020 Mar 8;12(3):617. doi: 10.3390/polym12030617 (PMC7182897; doi:10.3390/polym12030617)
Supplement: Supplementary file 1 [file polymers-12-00617-s001.pdf]

Supporting materials

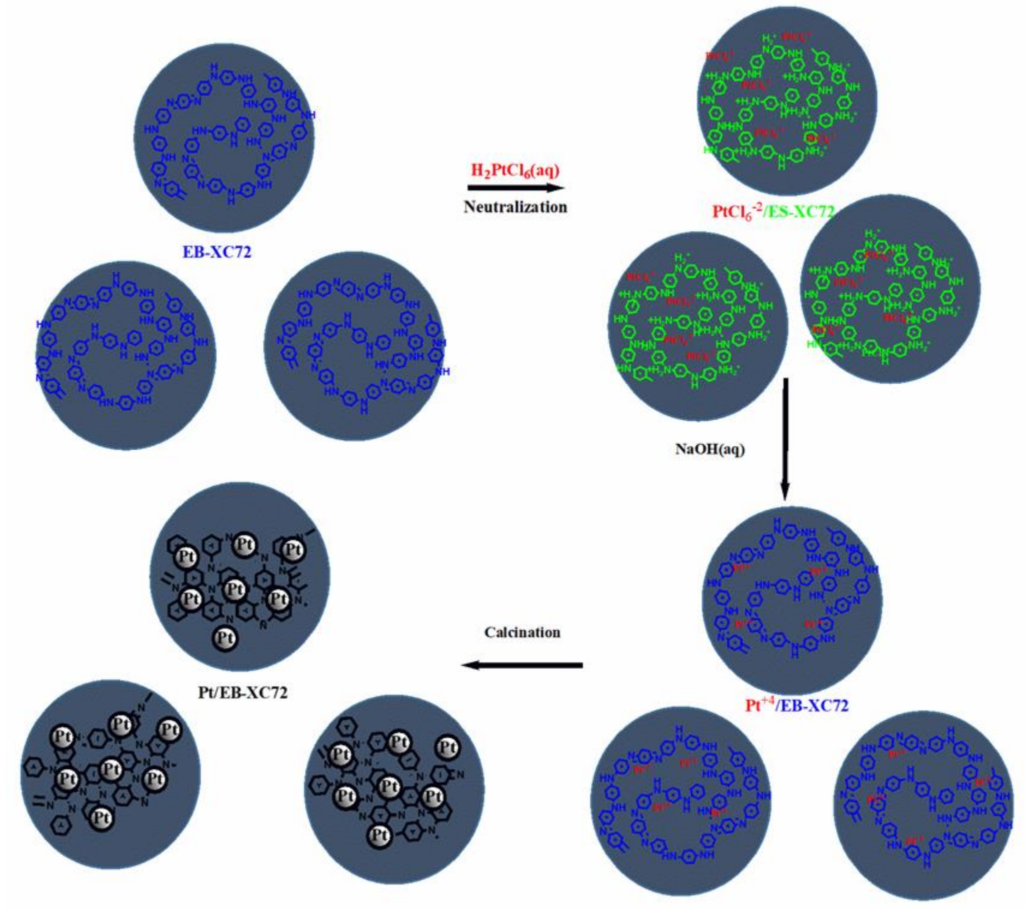

Figure S1. schematic diagram of the formation of Pt/EB-XC72 catalyst.

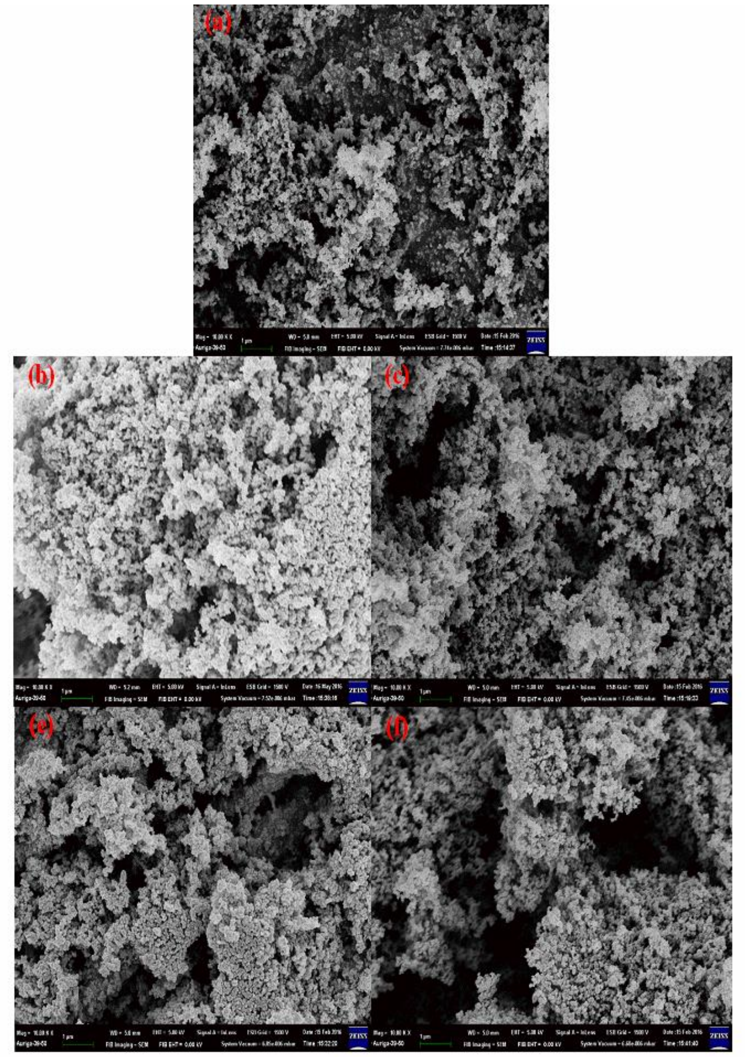

Figure S2. SEM images of Pt/XC72 treated at different temperatures.   neat XC72, (b) 500 ^{o}C, (c) 600 ^{o}C, (d) 800 ^{o}C, (e) 900 ^{o}C.

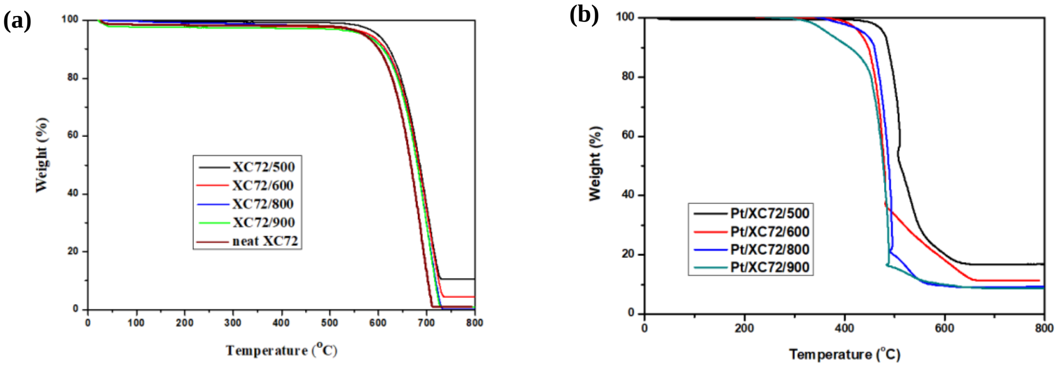

Figure S3. TGA thermograms of various XC72 treated at different temperatures without Pt (b) with Pt.
